# Challenges in Diagnosing Severe Mitral Paravalvular Leaks: A Case Report

**DOI:** 10.7759/cureus.84028

**Published:** 2025-05-13

**Authors:** Keitaro Yoshioka, Yota Kitagawa, Masaki Maekawa, Taro Kanamori, Ken Takemori

**Affiliations:** 1 Department of Anesthesiology, Kawaguchi Cardiovascular and Respiratory Hospital, Saitama, JPN; 2 Department of Cardiovascular Surgery, Kawaguchi Cardiovascular and Respiratory Hospital, Saitama, JPN

**Keywords:** acute kidney injury, congestive heart failure, hemolytic anemia, mitral valve evaluation, paravalvular leak, transesophageal echocardiography, transthoracic echocardiography

## Abstract

Symptomatic mitral paravalvular leaks (PVL) are a rare but potentially life-threatening complication after prosthetic valve replacement. While transthoracic echocardiography (TTE) is commonly used for diagnosis, it may miss PVL due to acoustic shadowing from prosthetic materials. We report a case of congestive heart failure with hemodynamic instability due to mitral PVL, which was not detected on TTE. Although transesophageal echocardiography (TEE) is the recommended for precise assessment, it was initially deferred in our case due to concerns that sedation and intubation required to perform TEE could worsen hemodynamic status without identification of the underlying cause. However, persistent heart failure and laboratory evidence of hemolysis ultimately prompted TEE, which revealed extensive mechanical valve dehiscence. Given the extent of the lesion, any further diagnostic delay could have been fatal. This case illustrates the diagnostic limitations of TTE and underscores the importance of early TEE in patients with prosthetic valves presenting with unexplained heart failure and hemolytic anemia.

## Introduction

The paravalvular leak (PVL) is defined as an abnormal flow around a prosthetic valve between adjacent heart chambers. Among PVLs, mitral PVL occurs in approximately 7%-17% of patients following valve replacement [1,2]. Although most mitral PVLs remain asymptomatic, approximately 2%-5% of mitral PVLs progress to symptomatic cases, leading to clinically significant complications such as congestive heart failure, hemolytic anemia, or infective endocarditis [1]. The pathophysiology of symptomatic PVL involves high-velocity regurgitant jets impacting prosthetic structures, which can induce hemolytic anemia and exacerbate heart failure. Transthoracic echocardiography (TTE) is typically the first-line modality for prosthetic valve evaluation, and several studies have reported that severe mitral PVL in patients presenting with heart failure was identified early on TTE, prompting emergency surgical intervention [3,4]. However, TTE has been reported to potentially miss significant mitral PVL, particularly due to imaging artifacts such as acoustic shadowing and flow masking caused by mechanical valves [5,6]. Indeed, the sensitivity, specificity, and diagnostic accuracy of TTE for detecting mitral PVL have been reported to be 81.3%, 95.6%, and 89.6%, which are lower than those of transesophageal echocardiography (TEE) at 96.2%, 95.8%, and 96.0% [7]. In the present case, TTE failed to detect the mitral PVL in a patient presenting with heart failure because of such artifacts, making diagnosis difficult and resulting in a delay in surgical intervention. In our case, TEE ultimately revealed the pathology, leading to definitive surgical intervention. This case underscores the diagnostic limitations of TTE and highlights the clinical scenarios in which TEE should be strongly considered, specifically in patients with a history of mitral valve replacement who present with refractory heart failure of unknown origin, particularly when accompanied by hemolytic findings or hemodynamic instability.

## Case presentation

A 60-year-old man with a history of mitral valve replacement had a mechanical valve placed for severe mitral regurgitation seven years ago. Two months before transfer to our hospital, he had presented to a previous hospital with dyspnea and was admitted for congestive heart failure management. TTE at that time revealed no definitive abnormalities. Despite medical treatment, his symptoms persisted, and he subsequently developed acute kidney injury. The patient was transferred to our hospital for further management of treatment-resistant heart failure and acute kidney injury.

**Figure 1:**
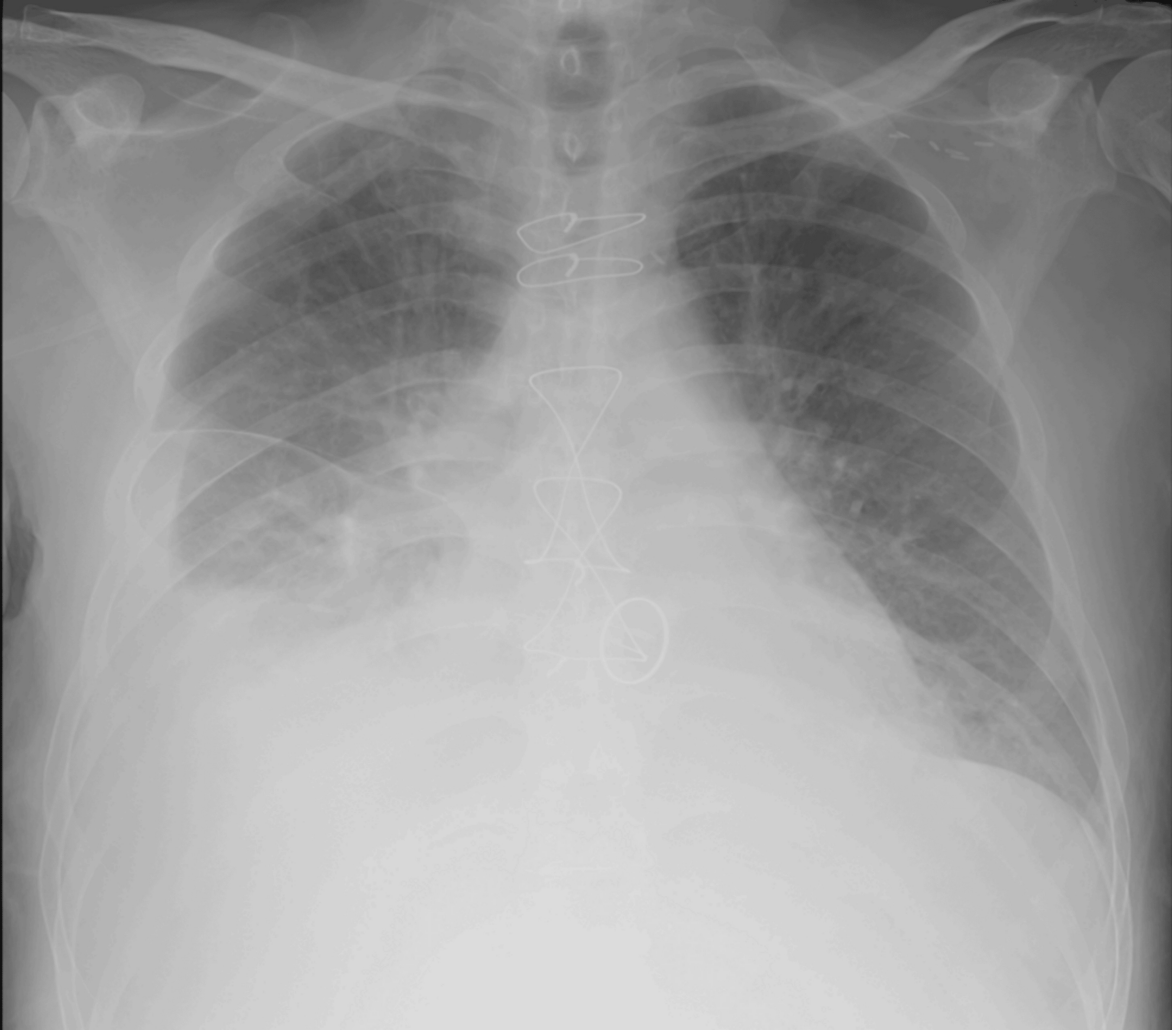
A chest X-ray image upon admission, demonstrating bilateral diffuse pulmonary opacities and a right-sided pleural effusion

Upon admission, the patient presented with orthopnea. There were no definitive auscultatory findings. His blood pressure was 71/38 mmHg, heart rate was 96 beats/minute, temperature was 35.9°C, and SpO_2_ was 99% on 2 L/minute of nasal oxygen. Laboratory findings on admission revealed that the blood sample was hemolyzed and showed anemia, elevated serum creatinine, and N-terminal pro-B-type natriuretic peptide (Table 1). Chest X-ray revealed bilateral diffuse pulmonary opacities and a right-sided pleural effusion (Figure 1). Although a right-sided pleural effusion was observed on chest X-ray, thoracentesis was not performed because the patient's respiratory status remained stable, and the effusion was not considered to require active intervention.

**Table 1:** Laboratory findings on admission The blood sample showed grade 1+ hemolysis. Elevated LDH levels were consistent with hemolysis. Additionally, the rise in BUN and serum potassium may reflect red blood cell breakdown and impaired renal impairment. NT-proBNP levels were markedly elevated, indicating worsening heart failure, which may have been further exacerbated by concurrent renal dysfunction The laboratory findings on admission show evidence of hemolysis and renal dysfunction, supporting the suspicion of PVL-related complications RBC: red blood cell count; Hb: hemoglobin; Ht: hematocrit; WBC: white blood cell count; EOS: eosinophils; LY: lymphocytes; MONO: monocytes; NE: neutrophils; BUN: blood urea nitrogen; Cr: creatinine; eGFR: estimated glomerular filtration rate; TP: total protein; Alb: albumin; AST: aspartate aminotransferase; ALT: alanine aminotransferase; γ-GTP: gamma-glutamyl transferase; LDH: lactate dehydrogenase; Na: sodium; K: potassium; CRP: C-reactive protein; TSH: thyroid-stimulating hormone; FT3: free triiodothyronine; FT4: free thyroxine; NT-proBNP: N-terminal pro-B-type natriuretic peptide; IU: international units

Parameter	Result and unit	Reference range
Hemolysis grade	1+	-
RBC	3.44 × 10⁶/µL	4.5-5.9 × 10⁶/µL
Hb	9.1 g/dL	13.5-17.5 g/dL
Ht	28.50%	38%-50%
WBC	7,660/µL (NE 83.1%, LY 7.2%, MONO 5.7%, EOS 3.9%)	4,000-10,000/µL
Platelet	35.5 × 10⁴/µL	15-40 × 10⁴/µL
BUN	96.9 mg/dL	8-20 mg/dL
Cr	6.22 mg/dL	0.61-1.04 mg/dL
eGFR	8 mL/minute/1.73 m²	>60 mL/minute/1.73 m²
TP	5.4 g/dL	6.7-8.3 g/dL
Alb	2.7 g/dL	3.8-5.2 g/dL
AST	46 IU/L	10-40 IU/L
ALT	20 IU/L	5-45 IU/L
γ-GTP	20 IU/L	<80 IU/L
LDH	526 IU/L	124-222 IU/L
Na	125 mEq/L	135-145 mEq/L
K	6.1 mEq/L	3.5-5.0 mEq/L
CRP	10.3 mg/dL	<0.3 mg/dL
TSH	0.87 µIU/mL	0.5-5.0 µIU/mL
FT3	1.86 pg/mL	2.3-4.2 pg/mL
FT4	0.83 ng/dL	0.8-1.9 ng/dL
NT-proBNP	12,505	<125 pg/mL

**Figure 2:**
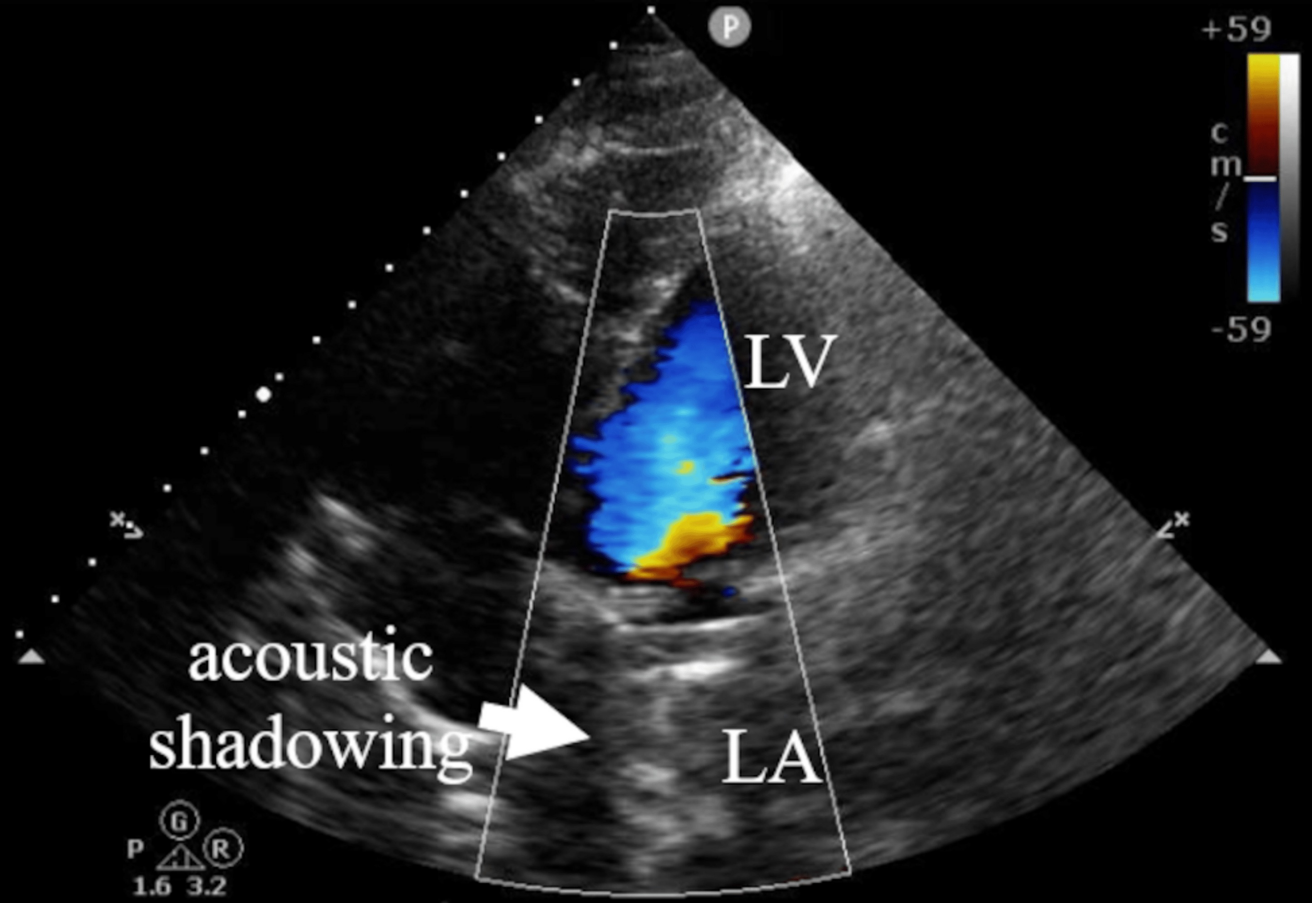
TTE at a four-chamber view showing the acoustic shadowing in the LA (white arrow). Color Doppler flow imaging reveals no definitive evidence of mitral paravalvular leak TTE: transthoracic echocardiography; LA: left atrium; LV: left ventricle

TTE showed a left ventricular ejection fraction of 74% and severe tricuspid regurgitation; however, there was acoustic shadowing in the left atrium and no definitive evidence of mitral PVL (Figure 2).

**Figure 3:**
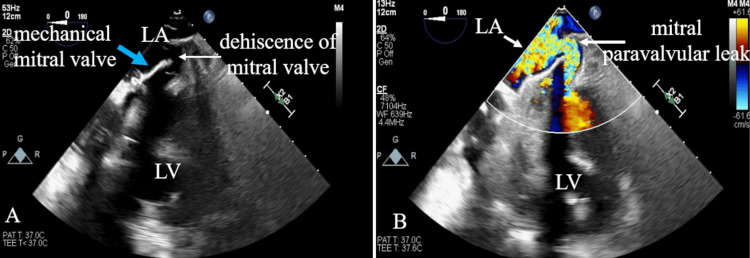
(A) 2D TEE at a four-chamber view showing mechanical mitral valve dehiscence indicated by the white arrow. (B) 2D TEE color Doppler flow imaging showing mitral paravalvular leak indicated by the white arrow. Based on these findings, more accurate anatomical assessment using three-dimensional TEE was considered 2D: two-dimensional; TEE: transesophageal echocardiography; LA: left atrium; LV: left ventricle

**Figure 4:**
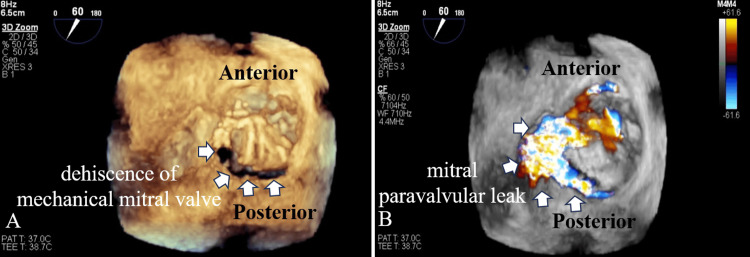
(A) 3D TEE of the mitral valve viewed en face from the left atrium showing mitral valve dehiscence on the posterior side (white arrow). (B) 3D TEE of the mitral valve viewed en face from the left atrium with color Doppler flow imaging showing mitral PVL (white arrow). These findings support the diagnosis of PVL as the underlying cause of refractory heart failure 3D: three-dimensional; TEE: transesophageal echocardiography; PVL: paravalvular leak

Pulmonary artery catheter revealed pulmonary hypertension, with a pulmonary artery pressure of 75/34 mmHg and a pulmonary wedge pressure of 39 mmHg. The differential diagnosis considered for the patient's refractory heart failure included volume overload due to renal impairment or beriberi heart disease. However, despite volume reduction via continuous hemodiafiltration and nutritional support, pulmonary hypertension and heart failure persisted. Moreover, TTE did not reveal definitive findings to explain the severity of heart failure. Therefore, TEE was performed under intubation to investigate the cause of pulmonary hypertension and to reassess the mechanical valve, due to treatment-resistant congestive heart failure complicated by hemolytic anemia. TEE, including two-dimensional and three-dimensional imaging, showed dehiscence of the mechanical mitral valve and mitral PVL (Figures 3, 4). The regurgitant jet was observed flowing from the dehiscence portion of the mechanical mitral valve into the left atrium (Figures 3B, 4B). Mitral PVL was identified as the cause underlying congestive heart failure and pulmonary hypertension.

Thereafter, emergency mitral valve replacement was performed. Although percutaneous closure can be considered an alternative to surgical mitral valve replacement, surgical intervention was chosen in the present case for the following reason: TEE revealed extensive mechanical valve detachment, which indicated that the patient was unsuitable for percutaneous repair. Therefore, open-heart surgery was necessary, as percutaneous closure was not indicated.

**Figure 5:**
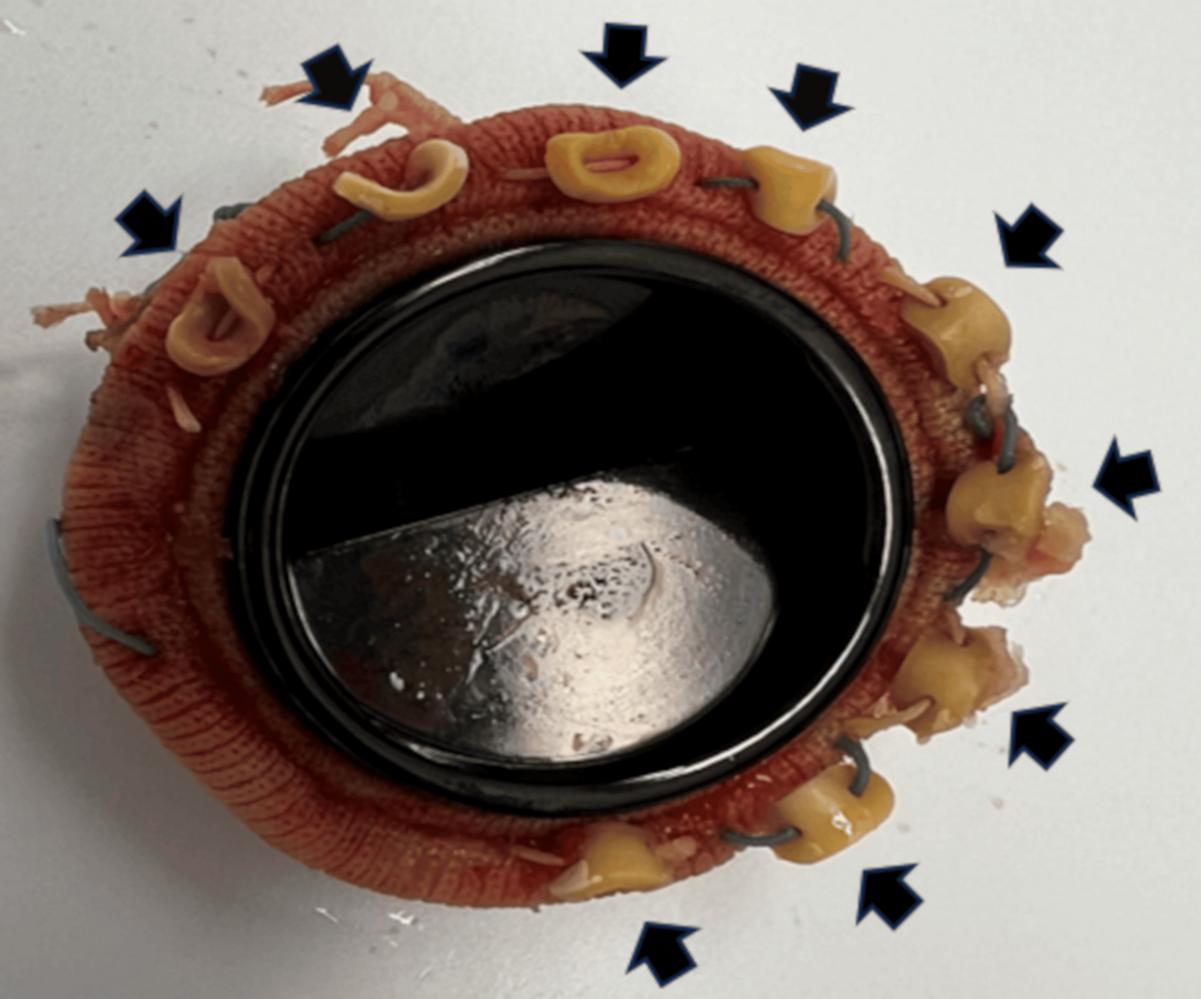
The detachment of three-quarters of the mechanical mitral valve Black arrows indicate that three-quarters of the sutures securing the mechanical valve had detached, along with surrounding tissue. No evidence of vegetation was observed

Intraoperative findings confirmed that approximately three-quarters of the prosthetic valve had detached, consistent with the preoperative assessment by TEE. No evidence of vegetation was observed (Figure 5).

The patient showed clinical improvement following intensive care unit management. He remained intubated for 18 days and underwent postoperative TEE, which revealed no significant abnormalities in prosthetic valve function. After a 52-day admission, he was ultimately discharged from the hospital without any complications.

The patient gave written and informed consent to the publication of this case report and all accompanying images.

## Discussion

Our case involved a patient with congestive heart failure due to a mitral PVL, which was detected by TEE but not identified on TTE. On admission, the patient presented with hypotension and severe pulmonary hypertension. The pulmonary hypertension was most likely caused by increased left atrial pressure and volume resulting from the PVL. This elevation in left atrial pressure was likely transmitted to the pulmonary circulation, subsequently leading to increased pulmonary arterial pressure. Additionally, the delay in detection of the PVL may have contributed to a cascade of hemodynamic deterioration, including reduced cardiac output, worsening renal perfusion, and progressive heart failure. In this case, earlier use of TEE might have facilitated earlier identification of the PVL and timely surgical intervention, potentially preventing further hemodynamic compromise.

Transthoracic echocardiography

TTE is typically the first-line imaging modality for evaluating valvular function. However, its diagnostic accuracy can be limited by technical constraints and imaging artifacts, particularly in patients with prosthetic valves. Furthermore, acoustic shadowing and flow masking caused by mechanical valves make the detection of regurgitation challenging, especially in the mitral and tricuspid positions [5,6]. In line with this, TTE failed to visualize mitral PVL in our case due to such artifacts, despite three-quarters of the mechanical mitral valve being detached (Figures 2, 5). These findings support the notion that TTE is insufficient to rule out prosthetic valve dysfunction due to prosthetic valve artifacts, particularly in the setting of mechanical valves.

Transesophageal echocardiography

TEE is recommended for detailed evaluation of cardiac structures, including the left atrial appendage, prosthetic valves, and paravalvular abscesses [8]. For quantitative assessment using specific Doppler findings, detecting proximal flow convergence (PISA) is useful in evaluating PVL [1]. While TTE has limitations in clearly visualizing PISA, TEE provides better imaging quality and may enable more accurate quantification. However, TEE is more invasive than TTE, often requiring sedation or tracheal intubation, which can limit its routine use. In our case, despite hemodynamic instability, the unclear etiology and ongoing clinical deterioration prompted the decision to perform TEE. The TEE clearly identified significant mitral valve dehiscence and mitral PVL, compared with no findings on initial TTE. Our case underscores the importance of considering TEE in patients with prosthetic valves who exhibit congestive heart failure with persistent hemodynamic abnormalities, even if there are no findings on TTE.

Nonechocardiographic imaging for PVL assessment

TEE remains the gold standard for diagnosing prosthetic valve dysfunction. However, other imaging modalities may play a complementary role, particularly when TEE is contraindicated or echocardiographic images are suboptimal. Among these, cardiac computed tomography (CT) has demonstrated superior diagnostic performance in detecting mitral PVL, with a sensitivity of 96.9% and a negative predictive value of 97.8%, compared to 81.3% and 87.8%, respectively, for TTE [7]. CT also enables accurate localization of PVL and evaluation of prosthetic valve structures. Cardiac magnetic resonance imaging (MRI) can visualize prosthetic valve regurgitation and allows quantitative assessment of mitral regurgitation volume and fraction [5]. Therefore, when echocardiographic findings are inconclusive or TEE is not feasible, cardiac CT and cardiac MRI should be considered valuable complementary imaging options.

Diagnostic challenges and clinical features of symptomatic PVL

In our case, although the patient presented with congestive heart failure, the diagnosis of PVL was challenging due to the absence of characteristic auscultatory findings and the lack of TTE findings. However, mild hemolysis and anemia revealed by laboratory tests suggested the presence of mitral PVL, with elevated levels of lactate dehydrogenase (LDH), blood urea nitrogen (BUN), and potassium (K). Hemolysis in PVL is thought to result from high-velocity regurgitant jets striking prosthetic structures, causing shear stress on the red blood cells [1,2]. Elevated LDH levels reflect hemolysis, as it is abundant in red blood cells and released upon their rupture. In addition, increased BUN and potassium levels may indicate red blood cell breakdown and associated renal impairment. In diagnostically challenging cases, such biochemical markers of hemolysis may serve as important clinical clues, prompting further evaluation with TEE when the etiology of heart failure remains unclear after initial noninvasive assessment.

Limitation

Previous studies have suggested that late-onset PVL may be associated with suture dehiscence due to infective endocarditis or with the resorption of incompletely debrided annular calcifications during the initial valve surgery [9]. In our case, the mitral valve replacement had been performed at another institution seven years before the current operation, making it difficult to determine the exact intraoperative findings or technical factors at that time. No obvious insufficiencies were noted during the current assessment, and therefore, no definitive etiology of the late-onset PVL was identified.

## Conclusions

We report a case of mitral PVL that was diagnosed only after performing TEE. While TTE was limited in visualizing prosthetic valves due to artifacts, TEE provided clear and definitive visualization of the valve dehiscence and regurgitation. In this case, TEE was initially deferred due to concerns that sedation and intubation required to perform TEE could further compromise the patient’s hemodynamic stability. However, the persistence of refractory heart failure and the presence of hemolytic anemia-an important clinical clue suggestive of mitral PVL-ultimately prompted the decision to proceed with TEE. After the operation, the patient showed clinical improvement and was discharged from the hospital without any complications after a 52-day admission. In patients with heart failure and a history of mitral valve replacement, routine clinical evaluation-including auscultation and TTE follow-up are essential. Although current guidelines recommend considering TEE when significant prosthetic mitral regurgitation is suspected, significant mitral regurgitation can occasionally be missed by TTE due to artifacts. Therefore, maintaining a low threshold for proceeding to TEE when minor clinical changes or hemodynamic instability are observed may help avoid diagnostic delays and facilitate timely intervention. This case highlights the importance of promptly considering TEE in such patients with unexplained congestive heart failure, particularly when TTE findings are absent and accompanied by hemodynamic instability or hemolytic anemia. It also highlights the risk of diagnostic delay and the need for clinician awareness of TTE limitations in prosthetic valve evaluation.
